# *Clostridium perfringens* Delta Toxin Is Sequence Related to Beta Toxin, NetB, and *Staphylococcus* Pore-Forming Toxins, but Shows Functional Differences

**DOI:** 10.1371/journal.pone.0003764

**Published:** 2008-11-19

**Authors:** Maria Manich, Oliver Knapp, Maryse Gibert, Elke Maier, Colette Jolivet-Reynaud, Blandine Geny, Roland Benz, Michel R. Popoff

**Affiliations:** 1 Bactéries anaérobies et Toxines, Institut Pasteur, Paris, France; 2 Lehrstuhl für Biotechnologie, Theodor-Boveri-Institut (Biozentrum) der Universität Würzburg, Würzburg, Germany; 3 Département Nouveaux Marqueurs, BioMérieux, Marcy l'Etoile, France; University of Minnesota, United States of America

## Abstract

*Clostridium perfringens* produces numerous toxins, which are responsible for severe diseases in man and animals. Delta toxin is one of the three hemolysins released by a number of *C. perfringens* type C and possibly type B strains. Delta toxin was characterized to be cytotoxic for cells expressing the ganglioside G_M2_ in their membrane. Here we report the genetic characterization of Delta toxin and its pore forming activity in lipid bilayers. Delta toxin consists of 318 amino acids, its 28 N-terminal amino acids corresponding to a signal peptide. The secreted Delta toxin (290 amino acids; 32619 Da) is a basic protein (pI 9.1) which shows a significant homology with *C. perfringens* Beta toxin (43% identity), with *C. perfringens* NetB (40% identity) and, to a lesser extent, with *Staphylococcus aureus* alpha toxin and leukotoxins. Recombinant Delta toxin showed a preference for binding to G_M2_, in contrast to Beta toxin, which did not bind to gangliosides. It is hemolytic for sheep red blood cells and cytotoxic for HeLa cells. In artificial diphytanoyl phosphatidylcholine membranes, Delta and Beta toxin formed channels. Conductance of the channels formed by Delta toxin, with a value of about 100 pS to more than 1 nS in 1 M KCl and a membrane potential of 20 mV, was higher than those formed by Beta toxin and their distribution was broader. The results of zero-current membrane potential measurements and single channel experiments suggest that Delta toxin forms slightly anion-selective channels, whereas the Beta toxin channels showed a preference for cations under the same conditions. *C. perfringens* Delta toxin shows a significant sequence homolgy with *C. perfringens* Beta and NetB toxins, as well as with *S. aureus* alpha hemolysin and leukotoxins, but exhibits different channel properties in lipid bilayers. In contrast to Beta toxin, Delta toxin recognizes G_M2_ as receptor and forms anion-selective channels.

## Introduction

*Clostridium perfringens* produces numerous toxins and is responsible for severe diseases in humans and animals including intestinal or foodborne diseases as well as gangrenes. Individual strains produce only subsets of toxins and are classically divided into five toxinotypes (A–E) based on their ability to synthesize Alpha, Beta, Epsilon and Iota toxins [1]. Delta toxin is one of the three hemolysins released by a number of *C. perfringens* type C and also possibly type B strains [2]. This toxin was purified from a *C. perfringens* type C strain and characterized as a basic (pI 9.1) 42 kDa protein which specifically hemolyzes erythrocytes from even-toed ungulates (sheep, goats and pigs) [2]. It was further showed that Delta toxin is cytotoxic for other cell types such as rabbit macrophages, human monocytes, and blood platelets from goat, rabbit, human and guinea pig [3]–[5]. The selective cytotoxicity of Delta toxin was correlated to a specific binding to the ganglioside G_M2_. Indeed, the hemolytic activity of Delta toxin as well as the binding of iodinated toxin to target erythrocytes is preferentially inhibited by G_M2_ [2], [6]. In addition, iodinated Delta toxin was shown to specifically bind to ganglioside G_M2_ extracted from membrane of sensitive cells and to liposome containing G_M2_ [7]. Thus Delta toxin was revealed to be an excellent tool for probing G_M2_ on cell membranes. In addition, Delta toxin selectively lyses malignant cells expressing G_M2_, such as carcinoma Me180, melanoma A375, and neuroblastoma C1300, and *in vivo* administration of Delta toxin to mice bearing these tumors significantly reduces tumor growth [8]. However, the mechanism of cytotoxicity remains unclear, since Delta toxin was reported to not insert into cell membrane and to induce membrane lysis by an unknown process [6], [7].

To further study the cytolytic mechanism of this toxin, we have cloned and produced a recombinant protein fully active on target red blood cells and which retains the binding to G_M2_. Here we report the molecular characterization and pore forming activity of the recombinant *C. perfringens* Delta toxin in lipid bilayer experiments in comparison with *C. perfringens* Beta toxin and *Staphylococcus aureus* alpha toxin, two well established pore-forming toxins. Channel formation by Delta toxin was more frequent than by beta toxin. Furthermore the conductance of the channels formed by Delta toxin was somewhat higher than those formed by Beta toxin and their distribution was broader. The results of zero-current membrane potential measurements suggested that Delta toxin formed slightly anion-selective channels, whereas the Beta toxin channels had a preference for cations under the same conditions.

## Results

### Cloning of the Delta toxin gene

Wild type Delta toxin was purified from *C. perfringens* strain CP24-03 as previously described [2] and submitted to microsequencing. Sequences of the 12 N-terminal residues as well as of two internal peptides were determined (Table 1). Oligonucleotide P723, deduced from internal sequence of peak 18, was synthesized according to the *Clostridium* codon usage and with inosine at the most degenerated positions (Table 1). This probe hybridized with total DNA as well as with plasmid preparations of *C. perfringens* strain CP24-03 and NCTC8131 (data not shown), suggesting that Delta toxin gene is located on plasmid DNA of these *Clostridium* strains. Furthermore, blast search did not reveal the presence of Delta toxin gene in chromosomal DNA from *C. perfringens* strains available in data banks. Plasmid DNA from strains CP24-03 and NCTC8131, and cut by *EcoR*I or *Hind*III showed different restriction patterns (data not shown), suggesting that they are not identical.

**Table 1:** Delat toxin peptides and oligonucleotide.

N-terminal	NDLGSKSEIRKE
Peak 16	EINSYHIAXDTEXQG
Peak 18	DGYNVNSWNIVYGNQMF
P723	AATTCITGGAATATIGTITATGGIAATCAAATGTT

Amino acid sequences of N-terminal and internal peptides of Delta toxin as well as oligonucleotide probe P723 complementary of peak 18 sequence according to the *Clostridium* codon usage.

**Figure 1:**
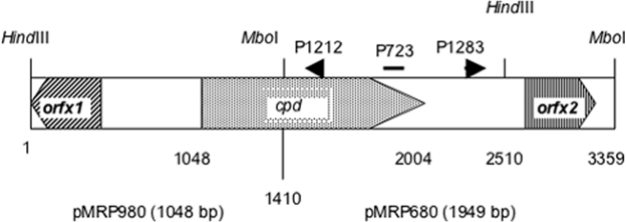
Delta gene organization in *C. perfringens* and cloning strategy of the Delta toxin gene. *C. perfringens* cloned DNA fragments into pCR2.1 yielding pMRP980 (insert of 1048 bp) and pMRP680 (insert of 1949 bp) are shown. P723 is an oligonucleotide deduced from an internal protein sequence (Table 1), P1212 and P1283 are the oligonucleotides designed for cloning the upstream part of *cpd* by inverse PCR.

The 2 kb *Mbo*I DNA fragment from *C. perfringens* 24-03 strain, recognized by P723, was cloned in pUC18 cut by *Bam*HI (pMRP680). The upstream part of Delta toxin gene was obtained by inverse PCR using the primers P1212 (5′- AACTATTTTTACAGAACTATC-3′) and P1283 (5′- ATGAAGGAATGGAAATAGATAAAAG-3′) (Figure 1) and plasmid DNA cut by *Hind*III. A 1,6 kb DNA fragment was amplified and cloned (pMRP980). DNA inserts of the two recombinant plasmids pMRP680 and pMRP980 were sequenced and assembled, and the 3359 bp DNA fragment containing the whole Delta toxin gene (Gene Bank accession number EU545552) is shown in Figure 1 and Figure S1.

### Features of the Delta toxin gene

The open reading frame from nucleotide 1048 to 2004 recognized by the probe P723 deduced from the Delta internal sequence was assigned to Delta toxin gene (*cpd*). A consensus ribosome binding site, GGGGTG, is located 7 nucleotides upstream of the initiation ATG codon (Figure S1). An inverted repeat of 15 nucleotides separated by 3 nucleotides and able to form a stem loop structure was identified 52 nucleotides downstream of the stop codon. This structure corresponds to a putative transcription terminator.

Downstream of *cpd* lies an open reading frame (orfx2) which is transcribed in the same orientation as *cpd* (Figure S1). Orfx2 encodes for a short basic protein of 198 aa (22552 Da and pI 8.86). At the amino acid level, Orfx2 shows significant identity level (26–31%) and similarity (44–47%) with transposase from various bacteria such as IS650/IS653 from *Bacillus holodurans*, IS116/IS110/IS902 family from *Desulfotomaculum reducens, Thermoanaerobacter tencogensis, Fervidobacterium nodosum, Clostridium kluveyri*, and *Clostridium cellulolyticum*, as well as transposase from *Fusobacterium nucleatum*.

Upstream of *cpd* (about 700 nucleotides) lies a partial open reading frame (*orfx1*) (Figure 1 and Figure S1), which encodes for a basic protein (112 aa, pI 10.53). The partial protein from *orfx1*, shows a strong identity (85%) with the chromosomal partitioning protein (PAR) identified in the *C. perfringens* genome [9]. Since *cpd* is flanked by two genes involved in DNA mobilization, it may be located on a mobile DNA element.

*Cpd* and *orfx2* genes PCR amplified and sequenced from strain NCTC8131 are identical to those of CP24-03. Thus, *cpd* sequence is the same in both strains.

### Delta toxin protein

The deduced Delta toxin protein is composed of 318 amino acids and has a predicted molecular mass of 35520 Da. N-terminal sequencing of the native Delta toxin (Table 1 and Figure S1) starts at Asn29 and matches residues Asn29 to Glu40. This indicates that the 28 N-terminal amino acids form a signal peptide, which is removed after secretion through the bacterial wall. In addition, this stretch of 28 amino acids contain residues characteristic of a signal peptide [10]: the presence of charged N-terminal residues at position 4 and 5 followed by a long hydrophobic core, of a turn residue (Pro25), and of a proteolytic cleavage Ala-Asn which is a common cleavage site for signal peptidase [11].

The secreted Delta protein contains 290 amino acids (32619 Da) and is predicted to be a basic protein (pI 9.15) in agreement with the experimentally determined pI of native Delta toxin (pI 9.1) [2]. However, the predicted molecular mass is lower than that of native Delta (42 kDa). The differences in the predicted and experimentally determined molecular masses of Delta toxin could be due to a particular conformational structure, as well as to a lack of technical precision in the previous determination. Internal peptide sequences of peak 18 and 16 (Table 1) exactly match residues 208-224 and 277-291, respectively (Figure S1), thus further confirming that the cloned *cpd* gene encodes for Delta toxin.

**Figure 2:**
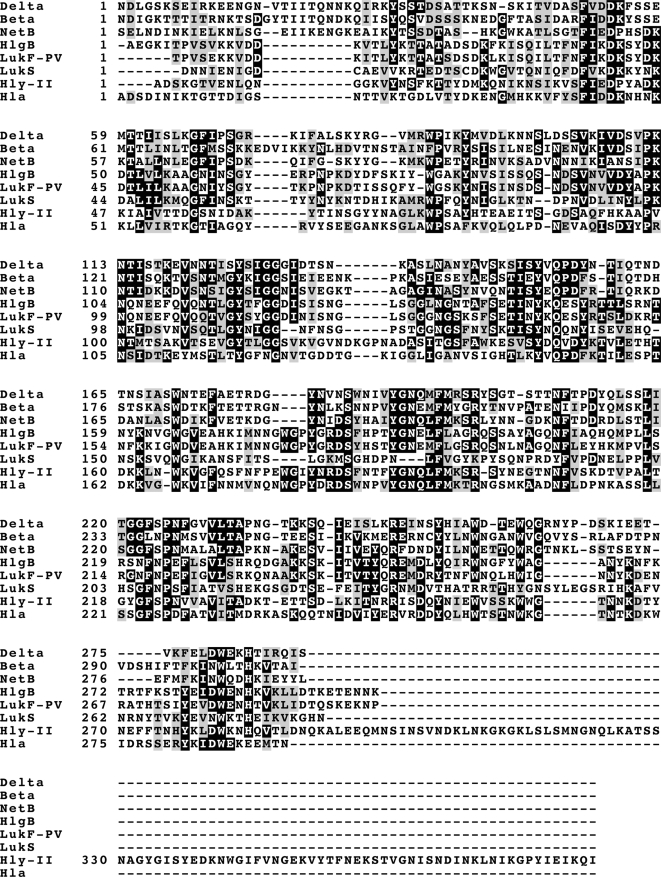
Amino acid sequence comparison of Delta toxin with other related toxins. Alignment of Delta amino acid sequence with those of *C. perfringens* Beta toxin (Beta; L13198), *C. perfringens* necrotic enteritis toxin B (NetB; ABW71134), *Staphylococcus aureus* gamma hemolysin (HlgB; YP041861), LukF-PV (ACB12456), LukS (X72700), *Bacillus cereus* hemolysin II (Hly-II; NP-833256), and *S. aureus* alpha toxin (Hla; X01645) using Clustal W (1.83) multiple sequence alignment program and Boxshade.

By comparison with protein sequences available in the data bank, Delta toxin displays significant homology with *C. perfringens* Beta toxin (43% identity, 63% similarity) (Figure 2). Beta toxin is produced by *C. perfringens* type B and C and is involved in necrotic enteritis in young animals and in humans (Pigbel and Darmbrand), as well as in sheep enterotoxemia. Beta toxin is synthesized as a 336 amino acid protein, the first 27 residues of which constitute a signal peptide. The secreted protein has a predicted molecular mass of 34861 Da and a pI of 5.5 [12]. Delta toxin is also significantly related (39.6% identity, 62% similarity) to *C. perfringens* necrotic enteritis toxin B-like (NetB), which has been recently identified in *C. perfringens* strains responsible for avian necrotic enteritis [13]. As found for Beta toxin and NetB [12], [13], Delta toxin is closely related, at the amino acid level, to pore forming cytolysins produced by other bacteria such as *Staphylococcus aureus* alpha-toxin (32% identity, 52% similarity), subunit F (LukF) and subunit D (LukD) (32% identity, 50% similarity), lukS (22% identity, 17% similarity) from *S. aureus* leukotoxin, Panton-Valentine leukocidin subunit F (LukF-PV) (31% identity, 50% similarity), components B and C from *S. aureus* gamma hemolysin (33% identity, 50% similarity) [14], [15]. In addition, Delta toxin is related to the hemolysin II from *Bacillus cereus* and *Bacillus thuringiensis* (identity 29%, similarity 49%) [16]. However, Delta toxin shows no significant similarity with *C. perfringens* Beta2 toxin [17], and cholesterol-dependent pore-forming toxins such as perfringolysin O and streptolysin O [18], [19].

### Recombinant Delta toxin

**Figure 3:**
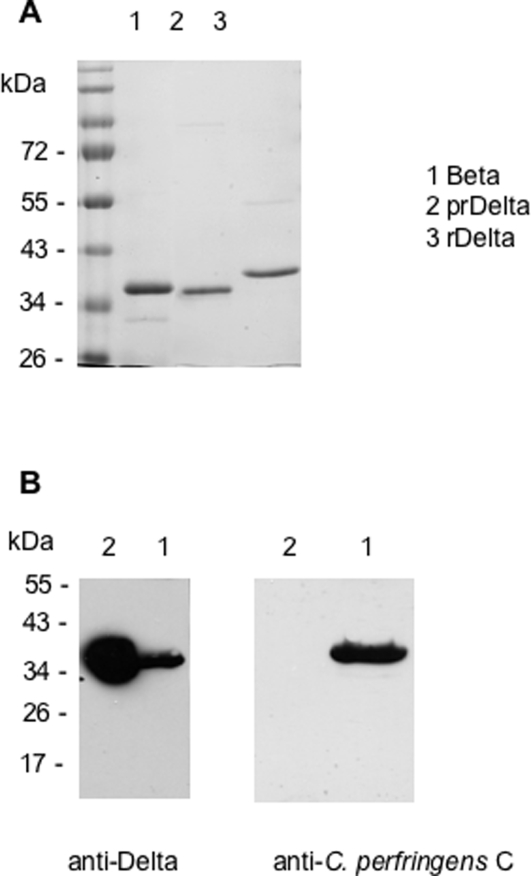
Recombinant Delta toxin and immuno-relatedness with *C. perfringens* Beta toxin. (A) SDS-PAGE stained with blue Coomassie R250 of *C. perfringens* Beta toxin (1), recombinant Delta toxin containing histidine tag (rDelta, 3), and rDelta processed by thrombin (prDelta, 2). (B) Immunoblotting of Delta and Beta toxins with anti-Delta toxin or anti-*C. perfringens* C. rDelta and Beta toxins were run on a SDS-PAGE, transferred on nitrocellulose, and immunoblotted with rabbit anti-Delta toxin or horse anti-*C. perfringens* C serum. Antibodies against Delta toxin recognized Delta toxin and to a lesser extent Beta toxin.

Recombinant Delta toxin (rDelta) without the signal peptide and with a N-terminal extension containing a six His-tag motif from the pET28a vector was produced in *E. coli* and purified on a cobalt column with elution buffer containing 100 mM imidazole. Processed recombinant Delta toxin (prDelta) was obtained after thrombin treatment. prDelta migrated at about 35–36 kDa on SDS-PAGE with a slightly higher molecular mass than that predicted (Figure 3A). rDelta and prDelta were recognized by antibodies raised against native Delta toxin as visualized by Western blotting, but not with anti-Beta antibodies (Figure 3 and not shown). However, immunopurified anti-Delta toxin antibodies interacted also with Beta toxin, although to a lesser extent than that with prDelta (Figure 3B). This indicates that Delta and Beta toxins share a low level of crossed immunological reactions.

### Hemolytic activity of recombinant Delta toxin

**Figure 4:**
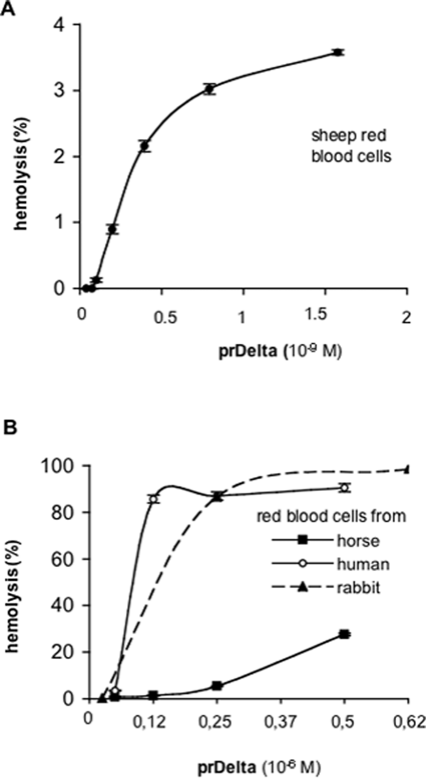
Hemolytic activity of recombinant Delta toxin. (A) Dose response of hemolytic activity of recombinant Delta toxin processed by thrombin (prDelta) with sheep red blood cells. (B) Hemolytic activity of prDelta with red blood cells from horse, human, or rabbit. Results are expressed as percentages of hemolysis from total hemolysis of red blood cells in distilled water (see Experimental Procedures). Data are means+/−standard deviations of two experiments performed in triplicate.

rDelta and prDelta were tested for hemolytic activity with red blood cells from various species. As previously found, sheep red blood cells were the most sensitive cells [2]. prDelta toxin was highly hemolytic towards sheep red blood cells, whereas rDelta toxin was inactive (data not shown), indicating that the N-terminal part is critical for the hemolytic activity and that a N-terminal extension such as that resulting from the cloning in pET28 and containing the six His motif impaired the hemolytic activity of Delta toxin. The 50% hemolytic concentration of prDelta toxin with sheep red blood cells was estimated to 10 ng/ml (0.25 nM) (Figure 4A), which is very close to that determined using native Delta toxin (5 ng/ml) [2]. prDelta was much less active on red blood cells from human (50% hemolytic concentration 0.1 10^−6^ M), rabbit (50% hemolytic concentration 0.12 10^−6^ M ) and horse (50% hemolytic concentration >0.6 10^−6^ M) (Figure 4B) in agreement with that already found with native Delta toxin.

**Figure 5:**
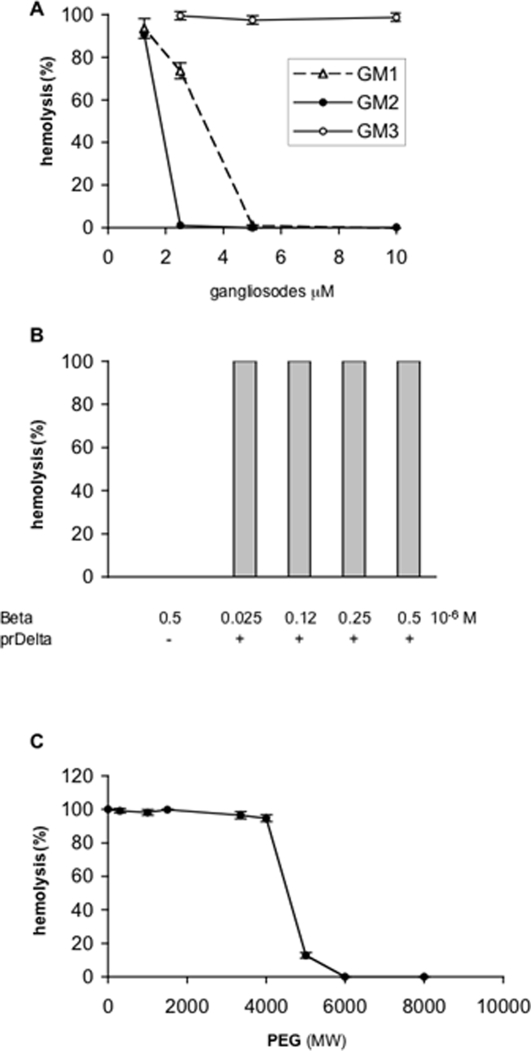
Inhibition of Delta toxin-induced hemolysis by gangliosides, PEG, but not by Beta toxin. (A) prDelta toxin (1.2 nM) yielding a 100% hemolysis of sheep red blood cells was incubated with increased concentrations of G_M1_, G_M2_ or G_M3_ for 10 min at 37°C prior to addition of red blood cells. Hemolytic activity was measured after further 45 min incubation at 37°C. (B) Sheep red blood cells were incubated with increased concentrations of Beta toxin prior addition of 1.2 nM prDelta. Data are means+/−standard deviations of two experiments performed in triplicate. (C) PEG of various MW ranging from 300 to 8000 were added in red blood cell suspension at 30 mM final concentration prior addition of prDelta (50 ng/ml).

As Delta toxin was reported to specifically bind to ganglioside G_M2_ [7], we tested the inhibition of Delta toxin hemolytic activity with various gangliosides. prDelta was incubated with gangliosides for 5 min and then tested for hemolytic activity with sheep red blood cells. G_M2_ efficiently inhibited the hemolytic activity of Delta toxin on sheep red blood cells (50% inhibiting concentration 2 µM), whereas G_M1_ was slightly less inhibitory (50% inhibiting concentration 4 µM) (Figure 5A). In contrast, G_M3_ in the same range of concentrations than those of G_M1_ or G_M2_, which were inhibitory, did not modify the hemolytic activity of prDelta (Figure 5A).

Since Delta toxin shows a significant sequence homology with Beta toxin, we checked whether both toxins competed for the same cell surface receptor. Beta toxin preparation up to 1.2 10^−6^ M incubated with *C. perfringens* type A serum (Wellcome) 1∶100 to neutralize trace of alpha toxin showed no hemolytic activity. *C. perfringens* type A serum did not inhibit the lethal activity of Beta toxin as tested in mice. The 50% lethal dose of the Beta toxin preparation was determined to be 150 ng per mice, in close agreement with previous results [20]. Sheep red blood cells preincubated with Beta toxin (0.025 to 0.5 10^−6^ M) and *C. perfringens* type A serum for 15 min at 37°C were completely lyzed with prDelta (1.2 10^−9^ M) as control (Figure 5B). Thus, Beta toxin did not recognize Delta toxin receptor on red blood cells.

PEG of various sizes were checked for inhibition of Delta toxin hemolytic activity (Figure 5C). These data are discussed below.

Beta toxin was found to be highly sensitive to trypsin. Biological activity of Beta toxin incubated with trypsin in a ratio of 1∶2 (w/w) was totally destroyed [21]. In contrast, Delta toxin is more resistant to trypsin. prDelta incubated with trypsin (1∶2; w/w) showed no significant loss of hemolytic activity with sheep red blood cells. A complete inactivation of prDelta was observed when incubated with higher trypsin ratio (1∶8) (data not shown).

### Binding of Delta toxin to gangliosides

**Figure 6:**
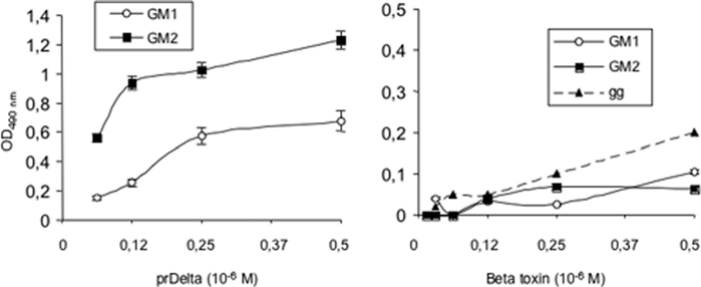
Binding of Delta toxin to gangliosides. Polystyrene microplate was coated with G_M1_, G_M2_ or a ganglioside mixture (gg) (1 µg per well) and incubated with increased concentrations of prDelta or Beta toxin. Bound toxin was monitored with specific antibodies against toxins and peroxidase conjugate as described in Experimental Procedure. prDelta toxin bound to G_M2_ and to a lower extent to G_M1_, whereas no significant Beta toxin binding to G_M1,_ G_M2_ or ganglioside mixture was detected. Data are means+/−standard deviations of two experiments in triplicate.

To further characterize the binding of Delta and Beta toxin to gangliosides, we used an ELISA assay. Microplates coated with G_M1_ or G_M2_ were incubated with prDelta or Beta toxin. As showed in Figure 6, prDelta bound to both immobilized G_M1_ and G_M2_ in a dose dependent-manner, and preferentially to GM2 versus to GM1. This agrees with previous findings showing a main interaction of Delta toxin with G_M2_ separated on thin-layer chromatography or inserted into liposomes [7]. In contrast, Beta toxin, in the same concentration range as prDelta, did not recognize G_M1,_ or G_M2_ or a ganglioside mixture in the ELISA assay (Figure 6).

### Binding of rDelta and prDelta to sensitive cells and oligomerization

**Figure 7:**
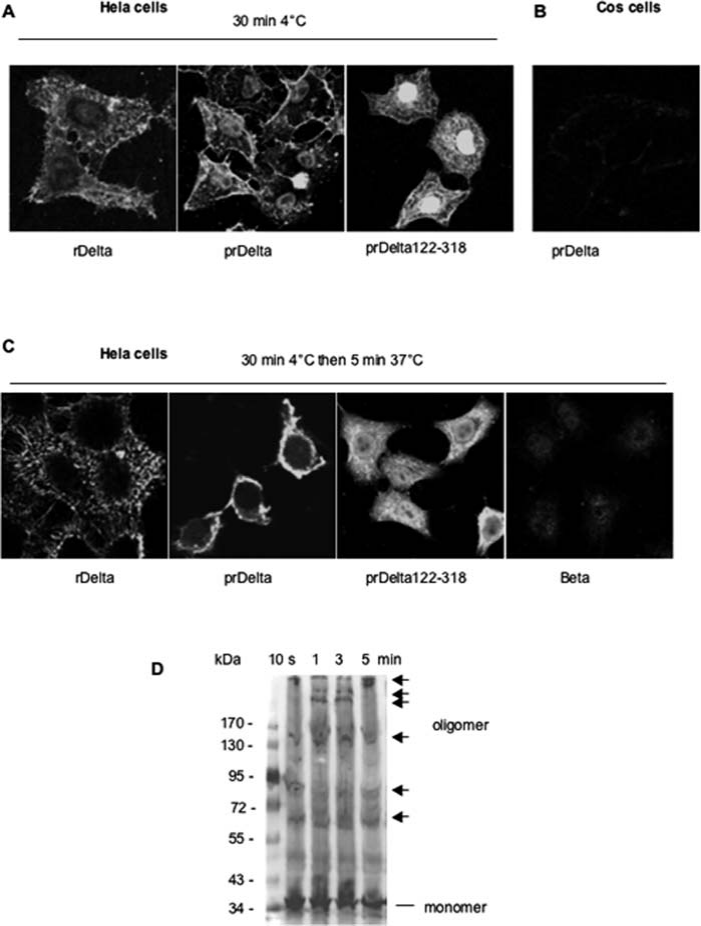
Binding of Delta toxin to HeLa cells. HeLa or Cos cells were incubated with toxin proteins at 4°C for 30 min, then washed (A) and in some experiments (B and C) cells were further incubated at 37°C in DMEM containing fetal calf serum for additional 5 min, and cells were finally processed for fluorescent microscopy. rDelta and prDelta122-318 bound to HeLa cells and did not induce any cell morphology alteration at 4° or 37°C (A and C). prDelta bound to HeLa cells and induced cell morphological alteration at 4°C and a complete cell rounding at 37°C (A and C), but did not bind to Cos cells (B). Beta toxin did not bind to HeLa cells (C). (D) Western blot analysis of prDelta oligomerization on HeLa cells over time at 37°C. HeLa cells were incubated for 10, 1, 2 or 5 min with prDelta (10 µg/ml) at 37°C, and then processed for Western blotting with anti-Delta toxin antibodies.

Since Delta toxin was shown to bind to and to be cytotoxic for GM2-expressing cells such as HeLa cells [8], we investigated the binding of rDelta and prDelta to cells by immunofluorescence. Toxin binding to cells was performed at 4°C for 30 min, the cells were then washed, and in some experiments they were incubated at 37°C for additional 5 min. rDelta and prDelta showed a staining of the periphery and surface of HeLa cells upon incubation at 4°C or 37°C (Figure 7A and C), whereas no significant immunofluorescence was evidenced in Cos cells at 4° or 37°C (Figure 7B and data not shown). In contrast to rDelta, which did not modify the morphology of HeLa cells, prDelta induced a cell rounding. HeLa cells treated with prDelta were smaller and rounded, indicating that prDelta was cytotoxic for HeLa cells (Figure 7C). Morphological alterations of HeLa cells treated with prDelta were observed at 4°C and were markedly more pronounced after incubation at 37°C (Figure 7A and C). It was suggested that the C-terminal domain of Delta toxin contains the binding activity to cells by using wild type Delta toxin treated with carboxypeptidase [7]. A truncated recombinant Delta toxin, corresponding to the C-terminal amino acids 122 to 318, was produced. The recombinant protein was treated with thrombin to remove the histidine tag (prDelta122-318). prDelta122-318 bound to HeLa cells incubated at 4°C or 37°C without inducing any cell morphological alteration (Figure 7A and C). A more diffuse staining was visualized in HeLa cells incubated with prDelta122-318 at 37°C compared to incubation at 4°C. In addition, prDelta122-318 was not hemolytic for sheep red blood cells, and preincubation of sheep red blood cells with a 100-fold excess of prDelta122-318 completely prevented the hemolytic activity of prDelta (data not shown). This supports that the 122-318 C-terminal part of Delta toxin contains the binding domain to target cells and that the N-terminal part is required for the cytotoxic activity. In contrast, Beta toxin showed no labeling of HeLa or Cos cells and induced no morphological change in these cells, but was cytotoxic for HL60 cells (Figure 7 and data not shown), in agreement with a previous work [22]. This supports again that Beta toxin did not recognize the same cell surface receptor as that of Delta toxin identified as G_M2_.

Then we checked whether prDelta forms oligomers in sensitive cells. HeLa cells were incubated with prDelta at 37°C for several time periods. As shown in Figure 7D, Western blot analysis of time-course studies revealed that prDelta rapidly formed large complexes in HeLa cells. After 1 or 3 min incubation, high MW bands corresponding to hexamers and heptamers were observed. Beta toxin was also found to form hexamers and heptamers in HL60 cells [22]. Further studies are needed nonetheless to determine the exact composition of Delta toxin oligomers.

### Inhibition of Delta toxin hemolytic activity by PEG

The precise mechanism of Delta toxin hemolytic activity has not yet been clearly defined. Delta toxin was found to bind rapidly and irreversibly to target cell membrane and then to induce a progressive leakage of intracellular compounds [8]. However, Delta toxin does not seem to insert into cell membrane as tested by toxin dissociation from membrane by chaotropic ions using a photoreactive probe [6], [7]. First, we checked whether Delta toxin hemolytic activity can be blocked by PEG from various MWs. As shown in Figure 5C, PEG5000 induced a 87% and PEG6000 a 100% inhibition of prDelta hemolytic activity with sheep red blood cells, whereas PEG of lower MWs were ineffective. This suggests that Delta toxin forms channels in red blood cell membrane which can be blocked by large MW compound. Delta toxin seems to form large pores in sheep red blood cells at least 4 nm in diameter based on the size of PEG5000 [23]. Such techniques of inhibition by PEG of various sizes have been used to estimate the pore size of several bacterial pore-forming toxins [24]–[28].

### Pore forming activity of Delta toxin and Beta toxin in lipid bilayers

Membranes were formed from 1% PC dissolved in n-decane. The addition of prDelta in small concentration (2.5 nM) to one or both sides of the lipid membranes resulted in a strong increase of conductance, whereas prDelta122-318 did not modify the lipid membrane conductance. This Delta toxin mediated conductance increase indicated that a receptor did not seem to be required for formation of ion-permeable channels by activated Delta toxin in black lipid bilayer membranes, similarly to what has been observed in the case of other membrane-active toxins [27], [29]. The conductance increase was not sudden but was a function of time after the addition of the protein to membranes in the black state. During the first 20 to 30 min, the membrane conductance increased by several orders of magnitude above that of membranes without the protein (from about 0.05 µS/cm2 to 150 µS/cm2). Only a small further increase (as compared with the initial one) occurred after that time.

**Figure 8:**
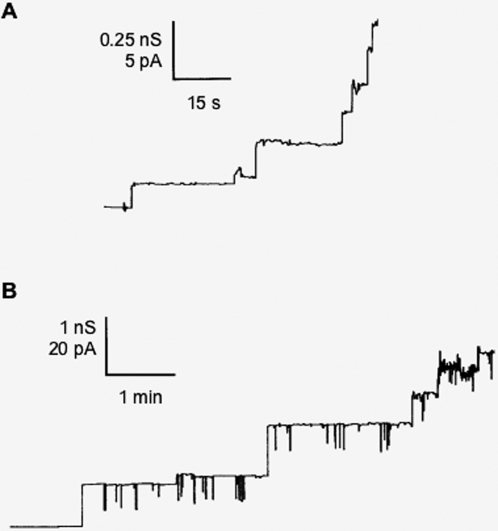
Pore forming activity of Delta toxin and Beta toxin in artifical lipid membranes. (A) Current recording of a diphytanoyl phosphatidylcholine/n-decane membrane after addition of 0.25 nM prDelta to the cis side of the membrane. The aqueous phase contained 1 M KCl, 10 mM Tris-HCl (pH 7). The applied membrane potential was 20 mV; T = 20°C. (B) Current recording of a diphytanoyl phosphatidylcholine/n-decane membrane after addition of 2.5 nM Beta toxin to the cis side of the membrane. The aqueous phase contained 1 M KCl, 10 mM Tris-HCl (pH 7). The applied membrane potential was 20 mV; T = 20°C.

**Figure 9:**
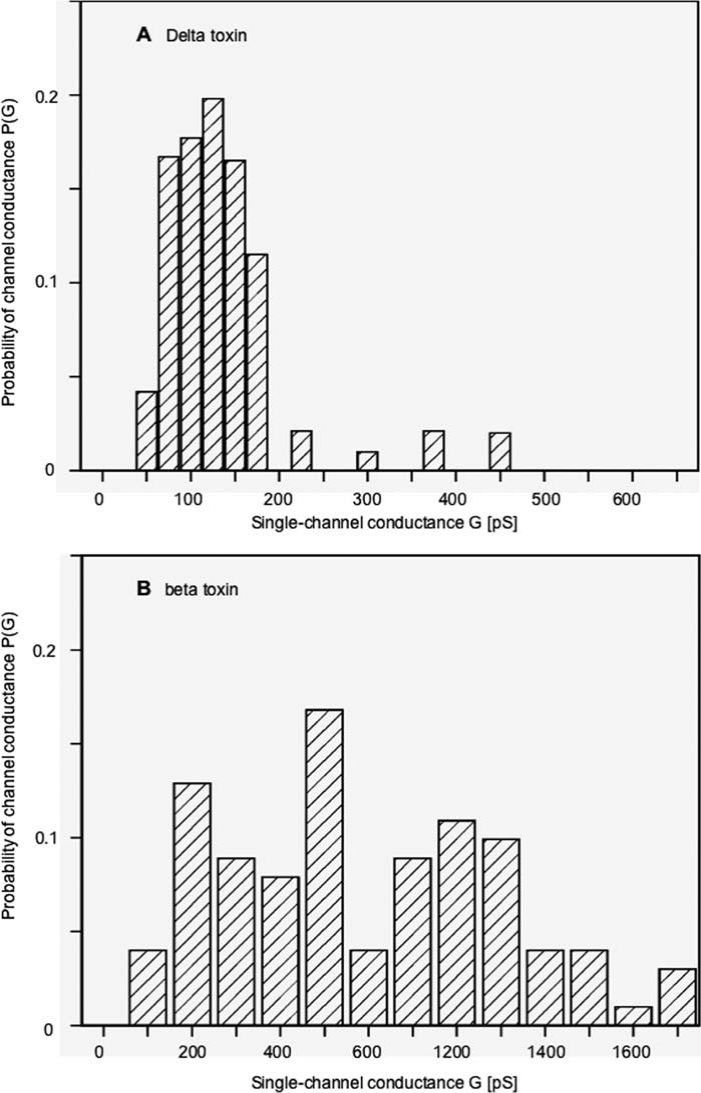
Properties of the pore-forming activity of Delta toxin and Beta toxin. Histograms of the conductance steps observed with diphytanoyl phosphatidylcholine/n-decane membranes in the presence of 0.25 nM prDelta toxin (A) and 2.5 nM Beta toxin (B) protein. The average single channel conductance was about 130 pS for 119 steps (panel A, Delta toxin) and about 550 pS for 143 steps (panel B, Beta toxin). The aqueous phase contained 1 M KCl, 10 mM Tris-HCl (pH 7) *T* = 25°C; *V_m_* = 20 mV.

The addition of smaller concentrations of Delta toxin (0.25 nM) to PC/n-decane membranes allowed the resolution of stepwise conductance increases. Figure 8A shows a single channel recording in the presence of Delta toxin added about 5 min after the membrane was in the black state. A few minutes after the addition of the protein, the current increased in the typical step-like characteristics, which is caused by the superposition of the long-lived Delta toxin channels. Figure 9 shows a histogram of the conductance fluctuations observed under the conditions of Figure 8A (20 mV membrane potential; 1 M KCl, 10 mM Tris-HCl, pH 7). Besides a major conductance step of about 125 pS (about 30% of all conductance fluctuations) we observed also channels with lower and higher single-channel conductance indicating some heterogeneity of the channels.

Delta toxin shares an interesting homology with Beta toxin (43% identity, 63% similarity). Therefore we performed also some lipid bilayer experiments to study its channel-forming properties in relation to that of Delta toxin. This toxin was also able to form channels in lipid bilayer membranes made of pure PC/n-decane. Interestingly, Beta toxin had a much smaller pore forming activity in membrane activity than Delta toxin under identical conditions (PC/n-decane membranes, 1 M KCl, 10 mM Tris-HCl, pH 7), which means that the number of channels was about 20 times smaller than those observed with the same concentration of Delta toxin. The conductance of the channels formed by Beta toxin was somewhat higher than that for prDelta under similar conditions and had a value of about 100 pS to more than 1 nS in 1 M KCl at membrane potential of 20 mV (see the single channel recording of Figure 8B). It is noteworthy that the distribution of channels was much more heterogeneous for Beta toxin than for Delta toxin. The histogram of channel conductance showed a broad histogram with maxima centered around 200 pS, 500 pS and 800 pS (see Figure 9B). The broad distribution of channels may be caused by the formation of channel aggregates of a unit conductance of about 200 pS. Because of their high molecular mass, these channel aggregates could have a much lower membrane activity as compared to Delta toxin.

Single-channel experiments with Delta and Beta toxin were also performed with salts containing ions other than K^+^ and Cl^−^. These experiments were done to get some insight in the biophysical properties of both toxins. The results summarized in Table 2 show that the Delta toxin channel is moderately anion selective. This conclusion can be derived from experiments in which KCl was replaced by LiCl or KCH_3_COO (see Table 2). The exchange of the mobile ions K^+^ and Cl^−^ by the less mobile ions Li^+^ and CH_3_COO^−^ indicated that replacement of K^+^ by Li^+^ had a minor influence on single-channel conductance. The effect of replacement of Cl^−^ by CH_3_COO^−^ was more substantial. The Beta toxin channels seem to be slightly cation selective because they had a much lower conductance in LiCl than in KCH3COO. These results were supported by zero-current membrane potential measurements, whereby 100 to 1000 prDelta or Beta toxin channels were reconstituted into the PC membranes. Then the KCl concentration on one side of the membranes was raised fivefold beginning from 100 mM and the zero-current potentials were measured 5 min after every increase of the salt gradients across the membrane. For KCl and prDelta the more dilute side of the membrane (100 mM) became negative and reached a potential of about −17 mV (mean value of 3 membranes) when the concentrated side was set at 500 mM KCl. In the case of Beta toxin, the more dilute side of the membranes became positive by about 16 mV (mean value of 3 membranes). Analysis of these membrane potentials using the Goldman-Hodgkin-Katz equation [30] confirmed the assumption of small anion selectivity of the Delta toxin channel and small cation selectivity of Beta toxin. The ratios of the permeability P_cation_ and P_anion_ were 0.35 and 2.6 for Delta toxin and Beta toxin, respectively.

**Table 2:** Average single-channel conductance, G, of Delta toxin and Beta toxin in different salt solutions.

Salt	Concentration [M]	G [pS]	
		Delta toxin	Beta toxin
LiCl	1.0	150	300
KCl	0.1	20	n. m.
	0.3	50	n. m.
	1.0	130	550
	3.0	250	n. m.
KCH_3_COO	1.0	30	450

The membranes were formed of 1% PC dissolved in n-decane. The aqueous solutions were buffered with 10 mM Tris-HCl and had a pH of 7. The applied voltage was 20 mV and the temperature was 20°C. The average single-channel conductance, G, was calculated from at least 80 single events derived from measurements of at least four individual membranes. n. m.: not measured.

## Discussion

Here we characterized *C. perfringens* Delta toxin gene and its translation in amino acid sequence. Delta toxin is one of the major toxins produced by *C. perfringens* which is lethal for mice and cytotoxic for various red and white blood cells [2], [4], [31].

Delta toxin contains a N-terminal 28 amino acid signal peptide as deduced from the predicted amino acid sequence and N-terminal sequencing of the secreted wild type toxin. This supports that Delta toxin is secreted from the bacteria through a signal peptide by the type II secretion system, as for the other *C. perfringens* toxins, except enterotoxin which lacks a signal peptide and is synthesized only during the sporulation phase (review in [32]. Deduced amino acid sequence of the secreted Delta toxin shows a strong homology with *C. perfringens* Beta toxin and NetB, as well as to a lower extent with *S. aureus* alpha-toxin and leukotoxins which have been characterized as pore-forming cytolysins [12], [13], [22], [33], [34]. In addition, blast search shows that Delta toxin retains a leukocidin domain. Thus Delta toxin seems to be part of pore-forming group of toxins encompassing *C. perfringens* Beta toxin and *S. aureus* pore-forming toxins [12] and probably other hemolysins like *Bacillus* hemolysins [16]. Moreover, residue R212 which has been found to be critical for the activity of *C. perfringens* Beta toxin, and its corresponding residue (R200) required for binding and oligomerization of *S. aureus* alpha toxin [35], are conserved in Delta toxin (R200) (Figure 2). Residues Y203, Y266, and W275, required for the full lethal activity, as well as D167 involved in protein conformation of Beta toxin [35], [36], correspond to Y191 Y253, W261 and D156 of Delta toxin respectively (Figure 2). However, Delta toxin differs from the other pore-forming toxins by its selectivity for only red blood cells and leukocytes from certain animal species. Indeed, Delta toxin was found to recognize gangliosides, preferentially G_M2_, as receptor on target cell surface [4], [6], [8]. As wild type toxin, recombinant Delta toxin was found to be cytotoxic for sheep red blood cells and HeLa cells which contain G_M2_ in their membrane [7], [8]. In addition, prDelta interacted directly with G_M2_ immobilized on polystyrene. But, it can not be ruled out that Delta toxin receptor is a dual receptor encompassing G_M2_ and another membrane component, such as a membrane protein, Despite, a significant homology at the amino acid sequence, Beta toxin was not cytotoxic for sheep red blood cells or HeLa cells, did bind neither to gangliosides G_M2,_ G_MI_, a ganglioside mixture nor to HeLa cells. Both Delta and Beta toxins could share a common mechanism of action involved in pore formation according to their sequence homology, but these toxins recognize distinct receptors on target cells. As previously suggested, the receptor binding domain lies in the C-terminal segment of Delta toxin [7]. Indeed, prDelta122-318 bound to HeLa cells as the whole recombinant toxin. Alignment of the C-terminal sequences of Delta and Beta toxins shows that the 66 C-terminal residues exhibit the lowest homology level (18% identity versus 42% for the whole sequences). This domain might contain specific binding site for the corresponding cell surface receptor.

Delta toxin is hemolytic and cytotoxic for sensitive red blood cells and other cells enriched in G_M2_ in their membrane by a non-defined mechanism [4], [7], [31]. Here, we show that Delta toxin forms channel in lipid bilayers comprised of PC. However, Delta toxin did not show a sharp maximum in single-channel conductance distribution. Instead the conductance was spread across a conductance range from about 75 to 175 pS in 1 M KCl. An even broader spectrum of channel conductance was observed with Beta toxin (from 100 to 1300 pS in 1 M KCl) with maxima at 200 pS, 500 pS and 800 pS. This is in qualitative agreement with a previous report showing a channel distribution from 10 to 380 pS in 100 mM NaCl with two major peaks of conductance at 60 and 110 pS [34]. Such a broad spectrum of conductance might be explained by insertion of several channels at the same time. Delta toxin seems to form smaller channels (4 times less) than Beta toxin considering the average conductance 130 pS compared to that of Beta toxin (550 pS in 1 M KCl), but we cannot exclude the possibility that the 500 pS channel represents already a channel oligomer. Another difference between Delta toxin and Beta toxin concerns the ion selectivity. Delta toxin exhibited weak anion selectivity as was found for *Staphylococcus* alpha hemolysin, epsilon toxin, and *C. septicum* alpha toxin [27], [37], [38]. In contrast, Beta toxin was cation selective (Table 2). Such an ion selectivity has already been reported for Beta toxin, which might account for the Beta toxin-induced perturbation in neuromuscular junctions [34].

The size and structure of Delta toxin channels remain to be determined. However, Delta toxin single channel conductance showed a reasonably narrow distribution with a mean value of 130 pS (Figure 9 and Table 2). In addition, the competition of Delta toxin-induced hemolytic activity with PEG of various sizes showed that inhibition occurred in a well defined manner with PEG molecular weight of 5000 and above (Figure 7B). This supports the suggestion that Delta toxin channels have a defined size, estimated to 4 nm in diameter based on the size of PEG5000. Thus, Delta toxin channels seem to be larger than those of *Staphylococcus* alpha hemolysin, the size of which is estimated to 2.8 nm in diameter by sugar exclusion methods [39], but which have a funnel shape with an entrance diameter of 2.8 nm decreasing to a minimum diameter of 1.4 nm at the bottom, depending upon the pore structure [38]. In comparison, *C. septicum* alpha toxin and aerolysin form channels with estimated diameter of 1.5 and 1 nm, respectively, in lipid membranes [24], [40]. However, Delta toxin channels might have a pore structure similar to that of *Staphylococcus* alpha hemolysin [38], which is considered as the basic model of β-barrel pore-forming toxins. The latter includes the aerolysin family which encompasses aerolysin, *C. septicum* alpha toxin, *C. perfringens* epsilon toxin, and probably Beta toxin [41]–[44]. The large channels formed by Delta toxin could account for the broad spectrum of conductance and a detergent-like effect. β-Barrel pore-forming toxins contain amphipatic β-hairpin forming sequences that associate to form a β-barrel when the toxin is oligomerized, which inserts itself into the lipid bilayer, resulting in pore formation [42]. Two stretches of alternating hydrophilic and hydrophobic residues were identified in the N-terminal region of Delta toxin (V84 to V110 and N120 to K148) and one in Beta toxin (S117 to I145) using the program http://psfs.cbrc.jp/tmbeta-net/. These sequences are likely two amphipatic β-strands involved in β-barrel formation. Only one of the two putative amphipatic β-hairpins in Delta toxin can be involved in pore formation as this was shown for *S. aureus* alpha toxin which requires one β-hairpin from each monomer to form the β-barrel [45]. Alternatively both putative amphipatic β-hairpins can be involved in pore formation as this is the case with the two transmembrane hairpins of Perfringolysin O [46], [47]. In contrast to aerolysin and probably *C. perfringens* epsilon toxin, where the β-hairpin forming the pore is located in domain 3 from the central region of the toxin [43], [48], the putative β-hairpins are found in the N-terminal part of Delta toxin. A similar location has been observed in *C. perfringens* enterotoxin, where residues from 81 to 106 predicted to form an amphipatic loop are important in pore formation [49]. However, further investigations are required to precisely define the pore domain in Delta and Beta toxins.

The role of Delta toxin in pathogenesis is not well understood. *C. perfringens* type B and C of which some strains can produce Delta toxin as an additional toxin, are involved in necrotic enteritis in various animal species, mainly piglets, and also in human (Pigbel). Beta toxin is considered as the main virulence factor of these strains [50], [51]. Recently, NetB was found to be responsible for necrotic enteritis in chicken using a *C. perfringens netB* mutant [13]. Based on the relatedness of Delta toxin with Beta and NetB toxins, Delta toxin might represent a potent virulence factor, which can induce intestinal diseases. The genetic characterization of Delta toxin will be useful for further epidemiological studies and mutant analysis to address the involvement of this toxin in pathology.

In conclusion, *C. perfringens* Delta toxin has been characterized at the amino acid level and is highly related to *C. perfringens* Beta toxin and to a lesser extent to *C. perfringens* NetB as well as to *Staphylococcus* alpha hemolysin and leukotoxins. As wild type toxin, recombinant Delta recognizes G_M2_ and is cytotoxic for cells enriched in G_M2_ in their membrane. The C-terminal part of Delta is involved in the recognition of the cell membrane receptor. Delta toxin forms channels in artificial lipid bilayers, which are anion selective and larger than those induced by *Staphylococcus* alpha hemolysin and toxins from the aerolysin family characterized by a heptameric pore structure. Delta toxin probably retains a common structure organization with that of β-pore forming toxins, but its exact mode of action remains to be determined. Since Delta toxin recognizes a specific receptor identified as the ganglioside G_M2_, this toxin and recombinant derivatives constitute valuable tools in cell biology and therapeutic agents, for example in identifying and treating cancer cells overexpressing G_M2_.

## Materials and Methods

### Protein microsequencing

*C. perfringens* Delta toxin was produced and purified as previously described [2]. Purified toxin was run on a 10% polyacrylamide gel containing 0.1% (wt/vol) sodium dodecyl sulfate (SDS) and transferred to a polyvinylidene difluoride membrane (Immobilon; Millipore). After amido black staining and destaining, the protein band was cut out and digested or not by trypsin. The peptides separated by high-performance liquid chromatography were microsequenced with a gas-phase protein sequencer (Applied Biosystems).

*C. perfringens* Beta toxin (lot SP3235) was a gift from Merial Company (Lyon, France).

### Bacterial DNA and plasmids

*C. perfringens* strains CP24-03 and NCTC8131 were grown in broth containing Trypticase (30 g/liter), yeast extract (20 g/liter), glucose (5 g/liter), and cysteine-HCl (0.5 g/liter) (pH 7.2) under anaerobic conditions. Clostridium genomic DNAs were extracted and purified as previously described and plasmid DNAs were prepared by the alkaline lysis method [52], [53].

Ligation, transformation and preparation of plasmid DNA from *E. coli* were conducted according to standard procedures [53].

### Production and purification of recombinant Delta toxin

The DNA coding for the full length Delta toxin without the signal peptide was PCR-amplified from strain CP24-03 with primers adding a *Nde*I site at the 5′ end and a *Sal*I site at the 3′ end. The PCR product was cloned into pCR2.1 vector (Invitrogen), and the digested insert with *Nde*I-*Sal*I was subcloned into pET28a (Novagen) at the corresponding sites. The recombinant protein was fused to an N-terminal extension containing a 6 histidine motif from pET28.

A recombinant plasmid expressing a truncated Delta protein was built by PCR amplification of DNA encoding Delta protein from D-122 to S-318 with primers adding a *Bam*HI site at the 5′ end and a *Eco*RI at the 3′ end and cloning into pET28a vector. Plasmid constructions were checked by DNA sequencing.

*E. coli* BL21 codonplus (DE3)-RIL (Stratagene) transformed with recombinant plasmids respectively were grown in LB medium containing kanamycine (50 µg/ml) at 26°C until a OD of 0,8. The expression was induced with IPTG (1 mM) and growth was continued overnight at 26°C. The bacteria were harvested by centrifugation, suspended in lysis buffer (50 mM Na_2_HPO_4_, pH 8, 300 mM NaCl, and protease inhibitors (Calbiochem), and sonicated. The cell debris were separated from the soluble fraction by centrifugation (18000g, 15 min). The soluble fraction was applied on a cobalt column (Talon, Qiagen). The column was washed with lysis buffer, and eluted with 2, 10 and 100 mM imidazole in the same buffer. The fractions containing highly purified recombinant proteins were pooled and dialyzed against 50 mM Na_2_HPO4, pH 8, 300 mM NaCl, 50% (vol/vol) glycerol. Cleavage by thrombin (Sigma) was performed by standard method.

### Antibodies

*Rabbit anti-*Delta toxin antibodies were prepared with purified native Delta toxin as previously described [2]. Immunopurified anti-Delta toxin antibodies were prepared as followed. Recombinant Delta toxin was run on SDS-PAGE, transferred onto nitrocellulose, and the band corresponding to Delta toxin (about 5 µg) was cut out, incubated in phosphate-buffered saline (PBS) containing 5% (wt/vol) milk and then overnight with a 10-fold dilution of rabbit anti-Delta toxin serum. Next, the nitrocellulose band was washed with PBS containing 0.1% (vol/vol) Tween 20, eluted with 0.1 M acetic acid and rapidly neutralized by addition of 0.3 M Tris HCl pH 8.8 final concentration. Eluted antibodies were used in Western blotting and detected with protein A peroxydase (Bio-Rad) and the chemiluminescence kit (Amersham Biosciences).

Horse serum anti *C. perfringens* C (Wellcome) and rabbit peroxidase antibodies against horse immunoglobulins (Sigma) were used for *C. perfringens* Beta toxin detection by Western blot. In some experiments mouse monoclonal antibody (a generous gift from B Kadra) was used.

### Hemolytic activity

Hemolytic activity was performed as previously described [2]. Erythrocytes from sheep, rabbit, horse, human blood were collected after centrifugation, washed in borate-buffered saline (BBS; 10 mM sodium borate, 150 mM NaCl, pH 8), and suspended (5% volume) in BBS. Delta toxin was serially diluted in BBS containing 0.1% (wt/vol) BSA, the final volume in each tube was adjusted to 1 ml. Then 0.5 ml of erythrocyte suspension was added to each tube. The tubes were incubated at 37°C for 45 min and then centrifuged (5000 rpm, 5 min). Optical density at 541 nm was measured in the supernatants. Hemolysis was expressed as percentage of the amount of hemoglobin released from 0.5 ml of 5% (wt/vol) erythrocytes suspended in 1.5 ml distilled water.

For hemolysis inhibition, gangliosides G_M1_, G_M2_, G_M3_ were prepared as stock solutions (10^−3^ M) in chloroform-methanol (2∶1), sonicated (10 s), and then diluted in BBS containing 0.1% (w/v) BSA. Delta toxin was added (50 ng/ml final concentration) in each tube containing various ganglioside concentrations. After incubation at 37°C for 10 min, 0.5 ml of erythrocyte suspension was added and the hemolytic assay was performed as described above.

### Biological activity

Swiss male mice, weighing 20–25 g, were used. Toxicity was determined by intraperitoneal injection of two-fold serial dilutions (0.5 ml of the toxin solution) into groups of four mice. Deaths occurring within 24 h were recorded.

### Binding to gangliosides

Binding to gangliosides was assayed by ELISA modified from Rummel et al. [54]. Ganglioside G_M1_, G_M2_ (Sigma-Aldrich) or a ganglioside mixture from bovine brain (Calbiochem) was dissolved in methanol and applied to 96-well polystyrene microtitration plate (Corning; 1 µg ganglioside in 100 µl/well). The solvent was evaporated at room temperature, and wells were washed three times with binding buffer (10 mM Tris-HCl, 10 mM Na_2_HPO_4_, 0.5% BSA (wt/vol), pH 7.2). Unspecific binding sites were blocked by incubating in PBS (10 mM Na_2_HPO_4_, 1.8 mM KH_2_PO_4_, 140 mM NaCl, pH 7.2) supplemented with 3% (wt/vol) BSA overnight at room temperature. Binding assays were performed in binding buffer (100 µl/well) containing serial dilutions of Delta or Beta toxin for 1 hr at room temperature. Unbound protein was removed by three washes in binding buffer. Bound toxin was detected by incubation with rabbit antibodies against Delta or mouse monoclonal antibody against Beta toxin in binding buffer for 1 hr at room temperature followed by three washes with binding buffer. Wells were then incubated with protein A-horseradish peroxidase (Bio-Rad) 1∶3000 or anti-mouse immunoglobulin peroxidase conjugate (Amersham-biotechs) in binding buffer for 1 hr at room temperature. Wells were washed three times with binding buffer and incubated in citrate buffer containing o-phenylenediamine and H_2_O_2_ (Sigma-Aldrich) as recommended by the manufacturer. Reaction was stopped by addition of HCl 3M, and the plates were read at 490 nm using a microplate reader (Bio-Rad).

### Immunofluorescence

Cells grown on glass cover slides were incubated with Delta or Beta toxin (20 µg/ml) for 30 min at 4°C and then washed with PBS. Cells were fixed with 4% (wt/vol) paraformaldehyde in PBS, washed with PBS, free radicals were quenched by incubation with 50 mM NH_4_Cl in PBS. Cells were permeabilized for 5 min with 0.2% (vol/vol) Triton X-100 in PBS, incubated with anti-Delta or Beta antibodies, and then with appropriate antibody coupled with Alexa488 for 45 min at room temperature. After washing in PBS, coverslips were mounted in Mowiol (Calbiochem), and observed by confocal fluorescence microscopy.

### Oligomerization of prDelta on cells

Oligomerization experiments were performed as previously described [55]. Briefly, HeLa cells (5×10^6^ cells per reaction) were incubated with prDelta (10 µg/ml), then washed three times with ice cold Hanks balanced salt solution (HBSS) containing 0.2% (wt/vol) BSA. The cells were lyzed with 100 µl RIPA buffer (25 mM Tris-HCl, pH 7.5, 150 mM NaCl, 1% (vol/vol) NP-40, 1% (wt/vol) sodium deoxycholate, 0.1% (wt/vol) SDS containing benzonase (3200 units/ml), DNase (1000 units/ml), RNase (1000 units/ml), protease inhibitors and phosphatases inhibitors for 15 min on ice. Cell lysates were loaded on a (0.1%) SDS-(10%) PAGE without heating and reducing agent in the sample buffer. Proteins were electrophoretically transferred onto nitrocellulose and probed with rabbit anti-Delta toxin and protein A conjugated to horseradish peroxidase (Bio-Rad). Immunoreactive bands were detected by using the ECL Western-blot system (Amersham).

### Black Lipid Bilayer Experiments

The methods used for black lipid bilayer experiments have been described previously in detail [56]. Membranes were formed from a 1% solution of pure diphytanoyl phosphatidylcholine (PC, Avanti Polar Lipids, Alabaster, AL) in n-decane. The experimental setup consisted of a Teflon chamber divided into two compartments by a thin wall and connected by a small circular hole for membrane formation with a surface area of about 0.3 to 0.5 mm2. Delta or Beta toxin was added to one side of the membrane, the cis side. Small potentials were applied to the membranes through Ag/AgCl electrodes (with salt bridges). The electrodes were connected in series to a voltage source and a homemade current amplifier made with a Burr Brown operational amplifier. The amplified signal was monitored on a storage oscilloscope (Tektronix 7633) and recorded on a strip chart (Rikadenki, Freiburg, Germany) or a tape recorder. Zero-current membrane potential measurements were performed by establishing salt gradients across membranes containing 100 to 1000 channels as has been described earlier [57].

## Supporting Information

Figure S1 Delta toxin gene (cpd) and flanking genes. Nucleotide sequence and amino acid translation of the Delta toxin gene and the flanking genes, orfX1 (upstream of Delta gene) and orfX2 (downstream of Delta gene) from C. perfringens CP24-03. The predicted Shine Dalgarno (GGGGTG) is underlined. The predicted signal peptide of Delta toxin is italicized. Delta peptides which have been sequenced by protein microsequencing are underlined with dashed line. Inverted repeat is indicated by arrows beneath the sequence. (GeneBank accession number EU545552).
(0.01 MB RTF)
