# Supplementary material for: Clostridium perfringens Delta Toxin Is Sequence Related to Beta Toxin, NetB, and Staphylococcus Pore-Forming Toxins, but Shows Functional Differences
Source: PLoS One. 2008 Nov 19;3(11):e3764. doi: 10.1371/journal.pone.0003764 (PMC2583947; doi:10.1371/journal.pone.0003764)
Supplement: Figure S1 — Delta toxin gene (cpd) and flanking genes. Nucleotide sequence and amino acid translation of the Delta toxin gene and the flanking genes, orfX1 (upstream of Delta gene) and orfX2 (downstream of Delta gene) from C. perfringens CP24-03. The predicted Shine Dalgarno (GGGGTG) is underlined. The predicted signal peptide of Delta toxin is italicized. Delta peptides which have been sequenced by protein microsequencing are underlined with dashed line. Inverted repeat is indicated by arrows beneath the sequence. (GeneBank accession number EU545552). (0.01 MB RTF) [file pone.0003764.s001.rtf]

1    AAGCTTTTGATAATCTATTTTGTTGACTACGGCTAACATCAAGTAGTATT
        A  K  S  L  R  N  Q  Q  S  R  S  V  D  L  L  I
51   TTTCTTTCTGCAAAAGCAAGGTTAACATTAGCTGGTAGTATATCAATATT
      K  R  E  A  F  A  L  N  V  N  A  P  L  I  D  I  N
101  TTCAAAATCAGTTTTCTTTATTACCTTATGTAAATCTAAATTCTTGTTTA
       E  F  D  T  K  K  I  V  K  H  L  D  L  N  K  N
151  AAAGAACATCAGCAATTGATAAATCTTCCATGCTATAGGCTTTAAATAGC
     L  L  V  D  A  I  S  L  D  E  M  S  Y  A  K  F  L
201  TTTGTTAAGTTAGATTGAGGGTCTAAGTCTACAAGTAATACTTTCTTTCC
      K  T  L  N  S  Q  P  D  L  D  V  L  L  V  K  K  G 
251  ATTTTGGCTTAAACAAGCTCCGAAGTTTGCTGTTGAAGTAGTTTTGGCAA
       N  Q  S  L  C  A  G  F  N  A  T  S  T  T  K  A
301  CTCCACCTTTAATATTAAAAATTGATAAGTTTTTCATCATAAATTCCTTC
     V  G  G  K  I  N  F  I  S  L  N  K  M
351  TTTCTTTTATAAATTTTATTTTTATGTCATGATGTCCCTAGGGGACACCT
401  AATAATTACTTTTTAAAAATTTCTATGTATCTGACTTTACGAAGAACTAA
451  TAAGTTTCCTTGTTTAACTAAATCTTCATAGCCCTTAGTTACATATTTTA
501  TAGTAAAGCCTTTAGTTCGGGATATCTTTaATTTTCATATCTCCCAATAA
551  ATCAACTCTAAGTTTAGCACCATTAGTAAGTTTATTAAATTGAGGTGCTA
601  AGAATAAATTATATTTTTTATTGACCATATAGATACCTCCTTCAAATTAA
651  GTAAACACTAGAGTTTAACACTTCGTGTTGTTGTTTTTTTGTTGAGCCTA
701  AGACTACACATAGGAGCGAATTGTAAAGGAAAATGGAGCAAAAAATTTTA
751  GCTGCTATGGAAGAATGGAATGGCCGATAAAAATTTTTGTGGAATGGCTT
801  TACAATTTTAGAGGATATGTGATAGGCTAGTGCCACAAAAAAACAGGAAC
851  TTAAAAAGAACTAGCCTAAGCTAGTTCTTACCTATTAGACTTATTAAATA
901  TTATGTGAGAGCGGAACAATAGTAAATTTAAAATGTAATATGAGAAGGAT
951  TTTATCAAATTTTTTTTTATTTTAATAAAAATCTGATATTTGTTTTGGAT
1001 GATTAATCATTATAATTAGATATTATAATGTAAAGGGGTGCTAAATTATG
                                                    M   1
1051 AATTCTAAAAAAATAATTACGACTATTTTATTATCTTCAATGGTAATAGC
     N  S  K  K  I  I  T  T  I  L  L  S  S  M  V  I  A  18
1101 TAATTTGGGGTTAGCTCAACCAGTTTTAGCAAATGATTTAGGGAGTAAAT
      N  L  G  L  A  Q  P  V  L  A  N  D  L  G  S  K  S 35
1151 CCGAAATAAGAAAAGAGGAAAATGGTAATGTTACTATTATAACTCAAAAT
       E  I  R  K  E  E  N  G  N  V  T  I  I  T  Q  N   51 
1201 AATAAACAAATAAGAAAATATTCTTCAACAGATTCAGCTACTACAAAAAG
     N  K  Q  I  R  K  Y  S  S  T  D  S  A  T  T  K  S  68
1251 TAATTCGAAAATAACAGTAGATGCTAGTTTTGTTGATGATAAATTTTCAT
      N  S  K  I  T  V  D  A  S  F  V  D  D  K  F  S  S 85
1301 CTGAGATGACAACAATTATTTCACTTAAAGGATTTATTCCATCAGGAAGA
       E  M  T  T  I  I  S  L  K  G  F  I  P  S  G  R   101
1351 AAAATATTTGCACTTTCTAAATATAGAGGTGTTATGAGATGGCCAATTAA
     K  I  F  A  L  S  K  Y  R  G  V  M  R  W  P  I  K  118
1401 ATATATGGTTGATCTTAAAAATAATTCATTGGATAGTTCTGTAAAAATAG
      Y  M  V  D  L  K  N  N  S  L  D  S  S  V  K  I  V 135
1451 TTGATAGTGTTCCTAAGAATACTATTTCAACAAAGGAAGTAAATAATACT
       D  S  V  P  K  N  T  I  S  T  K  E  V  N  N  T   151
1501 ATTTCATACTCTATAGGTGGAGGAATAGATACATCTAATAAAGCATCACT
     I  S  Y  S  I  G  G  G  I  D  T  S  N  K  A  S  L  168
1551 AAATGCAAATTATGCTGTTTCAAAATCAATAAGTTATGTACAACCTGATT
      N  A  N  Y  A  V  S  K  S  I  S  Y  V  Q  P  D  Y 185

1601 ATAATACAATACAAACTAATGATACGAATAGCATTGCTTCATGGAATACA
       N  T  I  Q  T  N  D  T  N  S  I  A  S  W  N  T   201
1651 GAATTTGCCGAAACGCGTGACGGATATAATGTAAATTCATGGAATATAGT
     E  F  A  E  T  R  D  G  Y  N  V  N  S  W  N  I  V  218
1701 ATATGGCAATCAAATGTTTATGAGAAGTAGATATTCAGGTACAAGTACTA
      Y  G  N  Q  M  F  M  R  S  R  Y  S  G  T  S  T  T 235
1751 CAAACTTTACTCCAGATTATCAATTATCATCTTTAATAACAGGAGGTTTT
       N  F  T  P  D  Y  Q  L  S  S  L  I  T  G  G  F   251
1801 TCGCCTAATTTTGGAGTGGTATTAACTGCGCCTAATGGAACAAAGAAATC
     S  P  N  F  G  V  V  L  T  A  P  N  G  T  K  K  S  268
1851 TCAAATAGAAATTTCTTTAAAACGTGAAATAAACTCATATCATATTGCAT
      Q  I  E  I  S  L  K  R  E  I  N  S  Y  H  I  A  W 285
1901 GGGACACTGAGTGGCAAGGTAGAAATTATCCAGATAGTAAAATTGAAGAA
       D  T  E  W  Q  G  R  N  Y  P  D  S  K  I  E  E   301
1951 ACAGTTAAGTTTGAACTTGACTGGGAGAAACACACTATAAGACAAATTTC
     T  V  K  F  E  L  D  W  E  K  H  T  I  R  Q  I  S  318
2001 TTAATTTAAAATTTTTTTTATTTACATACTTTTAAATTTGAAAACAATAT
      *  
2051 CTTAGATACAATAAGAGCATTAAAAATGCTCTTATTGTATACTATAGAAA

2101 ACTTAAGGAAGTTTATATAGATTATATTATTTAAAGAGTACTAGACTTTT
2151 ATTTAGTTAGTTAATATTAAATAAAACTTAGCCTTTCATCATTTTTCTTT
2201 TAACTTTCTATTTTATATTATTTAGCTTATCTTATACTAATTTTTGATTG
2251 AATTTCAAATCACATTTTTCTTTTCTTTCTTTTTTATTAGAACTTAATTT
2301 TCATGCAGTTTATCAATTAAAAATTAATATAGAATTAATAAATTTTAGGT
2351 GATAATATTTCCTTAAATTATAAAATATGGAGGAGATACTATGAGAATAT
2401 TTATAGGTATAGATATAGGGAAGGAATATAATTATGCGTCTTTTTTGAAT
2451 AATGAAGGAATGGAAATAGATAAAAGATTTAAGTTTAAGAATAATCATTT
2501 AGGCTTTCTAAGCTTTAAGGATAACATAGATAAGTTAACCTATGATTGTA
2551 ATTTTAACTATGATGATGTATTAATTGGATTTGAGTCTACTGGACATTAT
2601 TGGATAAATACAGATTGGTTTTTAACAGAAAAATTAAATATTAAAACTGT
2651 TATGGTTAGAAATGATGCTGTTAGGCATACTAGAGCTTTAAATTCACAAG
      M  V  R  N  D  A  V  R  H  T  R  A  L  N  S  Q  G
2701 GAAAAGGAAAAAATGATTCACTAGATAGTAGAACAATAGCAGAGTGTTTA
       K  G  K  N  D  S  L  D  S  R  T  I  A  E  C  L    
2751 AAGAATGGATATTACTTTGATGTCCAAGGAAGAAAAGAAGATTATATTGT
     K  N  G  Y  Y  F  D  V  Q  G  R  K  E  D  Y  I  V 
2801 ATTAAGACGTCTTACAAGAGAGAGAACTGAGTTAGAGAAAGAGATAGCAA
      L  R  R  L  T  R  E  R  T  E  L  E  K  E  I  A  R
2851 GATATAAAAATAGATATAGAGCATGGCTAGATGTCAATAATCAGATATTT
       Y  K  N  R  Y  R  A  W  L  D  V  N  N  Q  I  F  
2901 TTAGAAAACTTTTCAAATGTTTTTTCGACAGGAGCATTTTCACTATTAAA
     L  E  N  F  S  N  V  F  S  T  G  A  F  S  L  L  K 
2951 AGTATATCCATCATCTTTAGATATAATACAAGATAGTTATTTAGAAGTAA
      V  Y  P  S  S  L  D  I  I  Q  D  S  Y  L  E  V  R
3001 GAGAAAAGTTAAAAGAAGTAGGATATAAAAGAACTAATAATACTTTTAAG
       E  K  L  K  E  V  G  Y  K  R  T  N  N  T  F  K  
3051 ATTTATTATGAGGAAGCTAAAACATATAAAAATATAATTAATGGAATAAC
     I  Y  Y  E  E  A  K  T  Y  K  N  I  I  N  G  I  T
3101 TTATGCTGATAGATTAGAAATAAATGAGTACATAGAAACTTTAGAAATGT
      Y  A  D  R  L  E  I  N  E  Y  I  E  T  L  E  M  Y	
3151 ATATGAAGAAAAAAGAAAGTTTAGATGAAAAAATAGAAGCGTTGATGAAT
       M  K  K  K  E  S  L  D  E  K  I  E  A  L  M  N  
3201 TATTTAGAAGGCAAAGGTATATAATTCATTATCACAAATTAAAGGTATGC
     Y  L  E  G  K  G  I  *
3251 CAAAAGCACAAGTTGCTTCGCTTCTAGCTGAAATAGGAAATGTAAGTAAT
3301 TTTAAATCAGCTAGACATTTAATTAGTTATGCTGGACTAAATGTTCAAGG
3351 TGAAGGATC


Figure 1S
 
